# The Tupinambá of Maranhão State, Brazil, and medicinal plants described in the 17th century chronicles of the Capuchins Claude d’Abbeville and Yves d’Évreux

**DOI:** 10.1186/s13002-026-00872-x

**Published:** 2026-05-03

**Authors:** Warmiston Carvalho Gomes, Jairo Fernando Pereira Linhares, Maria Franco Trindade Medeiros

**Affiliations:** 1 Programa de Pós-Graduação em Ciências Biológicas (Botânica), Universidade Federal do Rio de Janeiro (UFRJ), Museu Nacional (MN), Rio de Janeiro, Brazil; 2 Departamento de Biologia, Universidade Estadual do Maranhão (UEMA), São Luís, Brazil; 3 Laboratório Interativo em Etnobotânica (LinE), Departamento de Botânica, Universidade Federal do Rio de Janeiro (UFRJ), Museu Nacional (MN), Rio de Janeiro, Brazil

**Keywords:** Indigenous knowledge, Historical ethnobotany, Medicinal flora, Missionary texts

## Abstract

**Background:**

Information concerning contact with the Tupinambá indigenous people and the flora in the state of Maranhão (Brazil) during the period of French occupation (17th century) is recorded in the chronicles of the Capuchin missionaries Claude d’Abbeville (?-1632) and Yves d’Évreux (1577–1632). These chronicles interrelate characters, spaces, environments, times, and events, based on the conceptual model of historical ethnobotany and memory. The aim of this work was to identify the vernacular names of medicinal plants found in the chronicles, considering a diachronic approach to investigate whether these medicinal species reported by the Tupinambá and mentioned by the missionaries are still present and used in the state of Maranhão today.

**Methods:**

The translated Portuguese versions of the chronicles “History of the Mission of the Capuchin Fathers on the island of Maranhão and their surroundings” and “Journey to Northern Brazil”, were analyzed. Information was extracted on medicinal plants and their uses, and botanical identifications were made based on similarities between vernacular names and morphological characteristics, medicinal indication, and area of ​​occurrence. Using scientific nomenclature, a search was made in databases and specialized literature on traditional uses.

**Results:**

Nine vernacular names were located in the chronicles, eight of which were identified. Fabaceae was the most represented family. Most identified species are native to Brazil (*n* = 6). Medical indications included, among others: digestive and dermatological problems, infections, and mood disorders. According to data found in the literature, some plants and their indications are still part of the traditional medicine systems in the current territory of the State of Maranhão.

**Conclusions:**

The documentary analysis of the information recorded by the missionaries in their chronicles more than 400 years ago highlighted the timelessness of the Tupinambá indigenous knowledge. This study brought to light elements of indigenous cultural heritage that can strengthen and value traditional knowledge about native flora.

**Supplementary Information:**

The online version contains supplementary material available at 10.1186/s13002-026-00872-x.

## Background

The French missionaries Claude d’Abbeville (?-1632) and Yves d’Évreux (1577–1632) of the Order of the Capuchin Friars Minor, were assigned to the northeastern coastal region of Brazil to help promote the colonial enterprise of Equinoctial France that was established in the current state of Maranhão in 1612. D’Abbeville lived in Maranhão for four months and d’Évreux for two years. During this period, they wrote chronicles describing aspects of the life of the Tupinambá indigenous people and the natural riches of the Island of Maranhão, including species of plants and animals [1–5].

The Tupinambá constitute one of the most important indigenous ethnic groups in Brazil, and belong to the Tupi macro-linguistic trunk, family Tupi-Guarani. Their culture is marked by complex and hierarchical social practices, power relations, vengeance, and a unique tribal cosmological religious system. They are also notable for the production of ceramics and artifacts, their subsistence practices, and plant domestication [6–13]. Following contact with Europeans in the late 15th century, the Tupinambá migrated to various regions of Brazil as a way to survive colonial expansion. Although once considered extinct, ethnic resurgence movements are currently spreading throughout the Brazilian territory [6, 14–16].

During the centuries of colonization (1500–1822), textual repertoires portrayed the Tupinambá and other indigenous ethnic groups. Examples include: *The Letter* of Pêro Vaz de Caminha (1500) [17]; *Letters from Brazil* (1549–1560) [18] by the Jesuit Manuel da Nóbrega; *Epistolary Narrative* (1583–1590) by Jesuit Fernão Cardim [19]; correspondences and writings (1554–1594) by father José de Anchieta [20]; *Two voyages to Brazil* (1557) by the German navigator Hans Staden [21]; *Singularities of Antarctic France* (1557–1558) [22], a book by the Franciscan André Thevet; and the *History of a voyage made to the land of Brazil* (1578) [23], in the format of chronicles by the Calvinist Jean de Léry; The *History of the Mission of the Capuchin Fathers on the Island of Maranhão and its Surroundings* (1614), a chronicle written by the Capuchin Claude d’Abbeville [24, 25]; and the *Journey to Northern Brazil made in the years 1613 to 1614 by Father Ivo d’Évreux* (published in 1874), a chronicle written by the Capuchin Yves d’Évreux [26], both have ethnographic and historical value regarding Tupinambá culture [2, 27–29]. In d’Évreux’s chronicle, one can perceive the continuation of d’Abbeville’s ethnographic narrative, especially in relation to indigenous Catholic conversions [3].

These works, by portraying different characters, highlight interethnic contacts, the cultural practices observed by Europeans, and the knowledge about nature, especially medicinal flora, shared by indigenous peoples [30–34]. Research into these records of Jesuit, Benedictine and Franciscan religious figures during colonial and imperial Brazil (1500–1889) reveals procedures adopted to identify, describe and understand the ecology and distribution of plants and animals, as well as the importance of these natural elements for human health [35–37].

The chronicles of d’Abbeville and d’Évreux highlight both the intellectual contribution of indigenous people and Capuchin missionaries in the analysis of patterns of use of medicinal plant during the colonial period of Brazil. The present research is grounded in two theoretical approaches. Following the premises of Medeiros [38, 39], historical ethnobotany acts as a mediator of the memories transcribed in these chronicles, enabling the correlation of characters at spatial, environmental, and temporal levels. In parallel with the concept of memory in oral history, as considered by Pollak [40], three constitutive criteria of these memories – events, characters, and places – reinforce the investigation of these chronicles. In this context, the objective of the present work was to identify the vernacular names of medicinal plants found in the chronicles of d’Abbeville and d’Évreux and, through a diachronic approach, to investigate whether these medicinal species reported by the Tupinambá and mentioned by these missionaries are still present and used in the state of Maranhão today.

## Methods

### Identification of the *corpus*

**Fig. 1:**
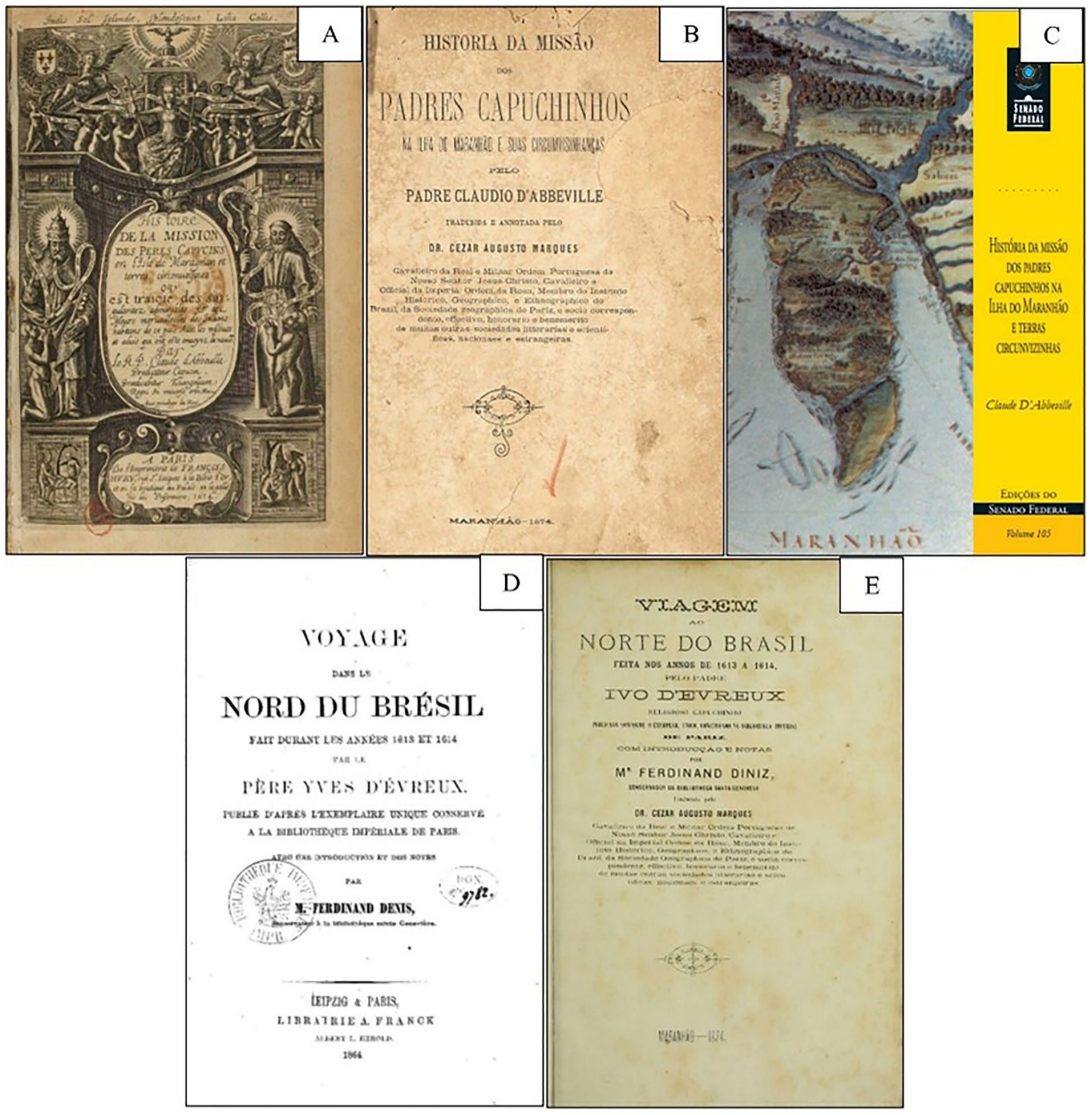
(**A**) *Histoire de la mission des Pères Capucins en l’Isle de Maragnan et terres circonvoisines*, by the Capuchin friar Claude d’Abbeville. Reproduction of page 2 of the original French edition from 1614. Source gallica.bnf.fr / *Bibliothèque nationale de France* [41]; (**B**) *History of the Mission of the Capuchin Fathers on the Island of Maranhão and Surrounding Lands.* Reproduction of page 3, published and translated into Portuguese by Dr. Cezar Augusto Marques in 1874. Collection of the *Biblioteca Digital do Senado Federal do Brasil* [24]; (**C**) *History of the Mission of the Capuchin Fathers on the Island of Maranhão and Surrounding Lands.* Reproduction of the cover, published and translated by Sérgio Milliet in 2008. Collection of the *Biblioteca Digital do Senado Federal do Brasil* [25]. (**D**) *Voyage dans le nord du Brésil fait durant les années et 1613 et 1614 par le Père Yves d’Évreux*. Reproduction of page 8, French-language edition from 1864. Source: gallica.bnf.fr / *Bibliothèque nationale de France* [42]; (**E**) *Viagem ao Norte do Brasil feita nos anos de 1613 a 1614 pelo Padre Ivo d’Evreux*. Reproduction of page 11, published and translated by Dr. Cezar Augusto Marques in 1874. Collection of the *Biblioteca Brasiliana Guita e José Mindlin* [26]

*History of the Mission of the Capuchin Fathers on the Island of Maranhão and its Surroundings*, authored by the Capuchin priest Claude d’Abbeville, as originally published by François Huby in 1614 in Paris and consisting of 841 pages, was consulted in its digital version on the Gallica digital platform (https://gallica.bnf.fr) of the *Bibliothèque nationale of France* (Fig. 1A). Two digital facsimile versions in Portuguese, were chosen for use. One version was published in 1874 [24], comprising 62 chapters and 456 pages (Fig. 1B); another updated version was published in 2008 [25] (Fig. 1C). Both are public domain translations and have been made available by the *Biblioteca Digital do Senado Federal do Brasil* (https://www12.senado.leg.br/institucional/biblioteca).

After an in-depth reading, in *Chapter XXXVIII – On the things that are ordinarily found on the Island of Maranhão and its surroundings*,* and first of all the fruit trees* [24, 25], accounts referring to the vernacular names of medicinal plants were identified. Comparisons were made between the translations, seeking similarities and differences among the descriptions of plant uses.

*Journey to Northern Brazil made in the years 1613 to 1614 by Father Yves d’Évreux*, written by the Capuchin Friar Yves d’Évreux, is a unique copy preserved at the *Bibliothèque Impériale de Paris*. The work includes an introduction and notes by M. Ferdinand Deniz, curator of the *Bibliothèque Saint Geneviève*. It was edited by *Librairie A. Franck* in Leipzig in 1615 but only published in 1864. The edition comprises 566 pages and was consulted in its digital version available on the Gallica online platform of the *Bibliothèque nationale de France* (Fig. 1D). A digital facsimile version translated into Portuguese was used [26]. A public domain version from the digital collection of the *Biblioteca Brasiliana Guita e José Mindlin* (https://www.bbm.usp.br/pt-br/), published in 1874 in Maranhão by Typografia do Frias, consisting of 64 chapters spread across 427 pages (Fig. 1E).

### Data collection and analysis

**Fig. 2:**
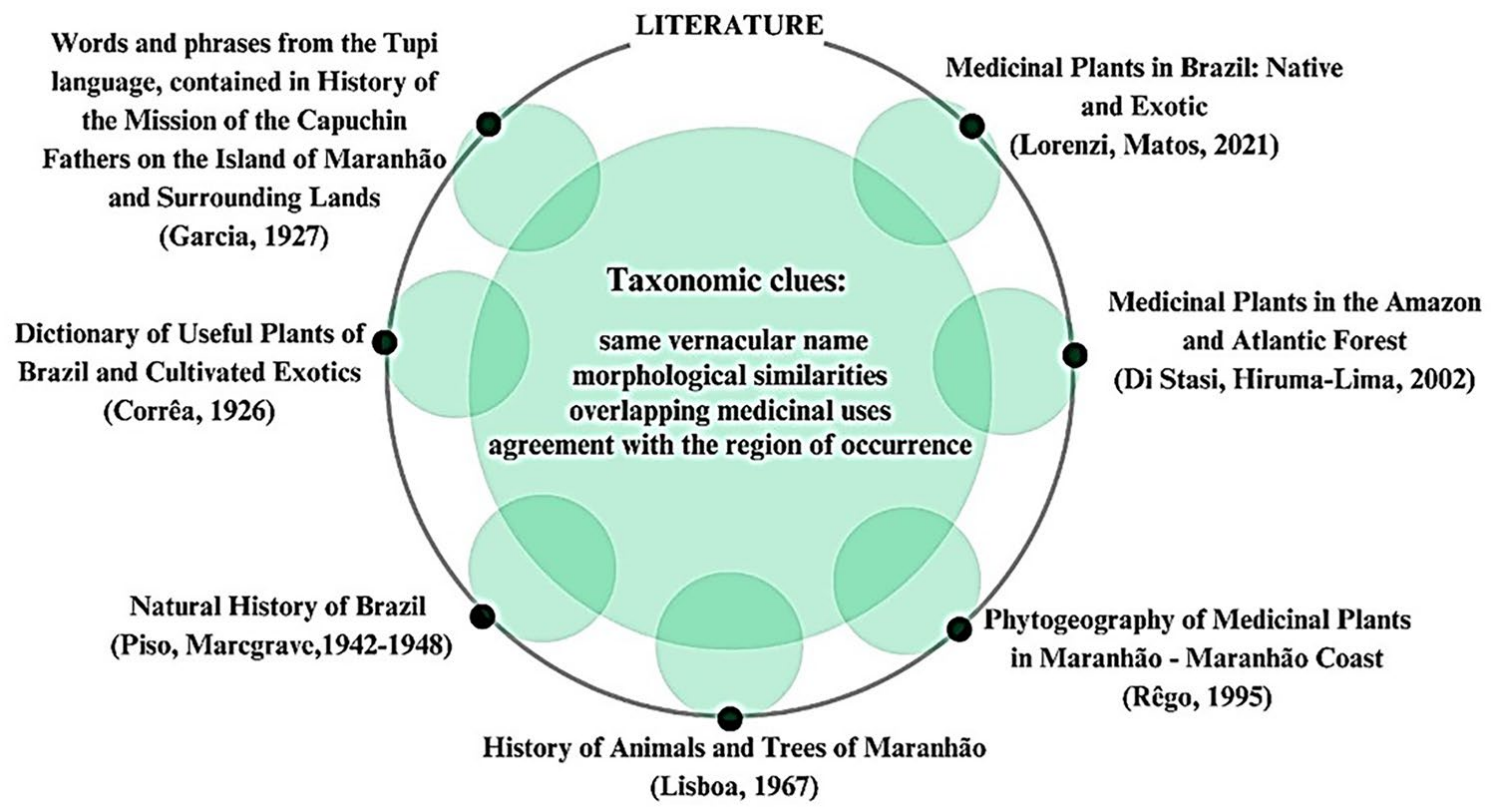
Literature consulted and taxonomic clues used to identify vernacular names [44–51]

The vernacular names found in the chronicles were coded as medicinal plants and maintained in their original paleographic form [43]. Similarities between the descriptions of medicinal plants and their taxonomic clues were considered for botanical identifications by means of comparisons with information found in the technical literature (Fig. 2) [44–51]. To confirm the botanical nomenclature, geographic distributions, and origins, the *Flora e Funga do Brasil* [52], Plants of the World Online [53], and Medicinal Plant Name Services [54] were consulted.

To understand the terms related to the diseases mentioned in the chronicles, their descriptions were compared to those in the *Diccionario de Medicina Popular e das sciencias accessórias para uso das familias* (Dictionary of Popular Medicine and Accessory Sciences for the Use of Families) [55] and the *Formulário e Guia Médico* (Medical Form and Guide) [56], both popular medical manuals by Dr. Chernoviz.

The construction of a correlated memory between these characters and their spatial, environmental, and temporal contexts was observed through the diachronic analysis of medicinal use by consulting virtual herbaria and textual productions concerning the flora of Maranhão.

**Fig. 3:**
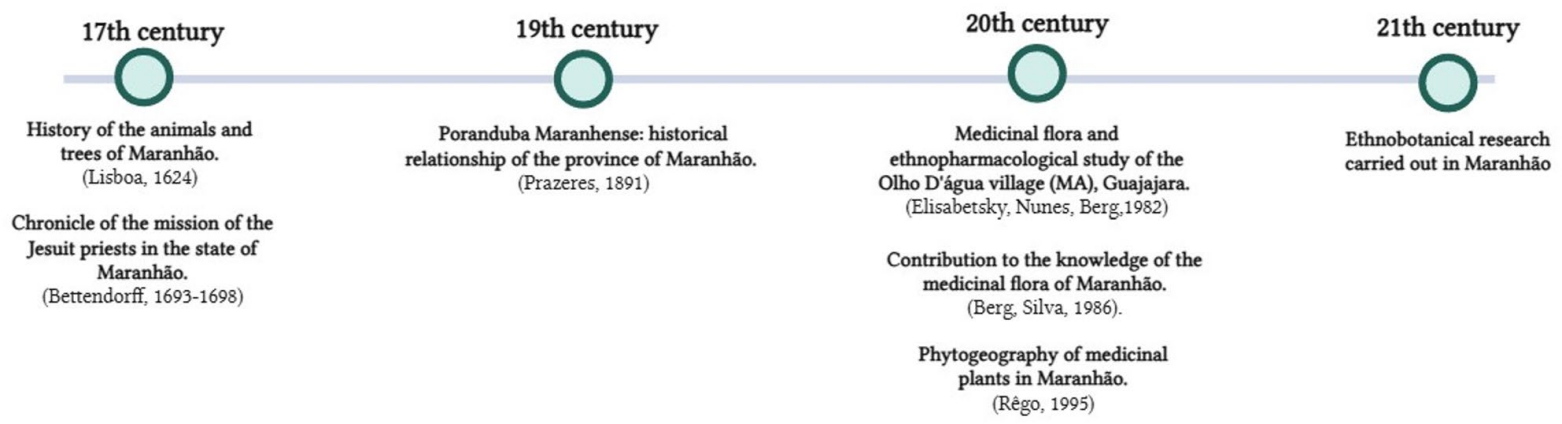
Temporal panel of the textual corpus used to verify the timelessness of medicinal uses of species utilized by the Tupinambá of Maranhão (Brazil), as mentioned by Claude d’Abbeville [24, 25] and Yves d’Évreux [26]

The textual material concerning the set of medicinal species from the 17th to the 21st century was selected and organized along a timeline beginning with the chronicles of Claude d’Abbeville [24, 25] and Yves d’Évreux [26] (Fig. 3). Two temporal subsets were considered: (1) the 17th, 19th, and 20th centuries, based on documentary records and botanical works available in physical or digital format with historical and scientific relevance for Maranhão [48, 49, 57–60]; (2) the 21st century, based on 20 ethnobotanical studies [61–80] selected through searches in the Google Scholar, Scielo, and PubMed databases using the terms “Maranhão”, “medicinal plants” and “ethnobotany”, published between 2002 and 2025. Exclusion criterion adopted: Studies that did not mention the uses of the medicinal plants found in the chronicles.

The SpeciesLink [81] and GBIF [82] platforms were consulted using the scientific names of medicinal plants combined with the filter “Maranhão”. The selection of collections with botanical records was based on: (1) presence of information on medicinal uses; and (2) geographic location, covering the current regions of the state of Maranhão. This survey was carried out between 2024 and 2026.

## Results and discussion

Claude d’Abbeville [24, 25] mentioned five medicinal plants with medicinal indications, of which four were able to be identified at the family and species levels. The botanical identification of the vernacular name “usenpopuytan” has not been reached. Possibly, the missionary was referring to some tuberous species of the genus *Maranta* [83–85]. Yves d’Évreux [26], recorded four medicinal plants, identified at the family and species levels (Table 1). Other species and uses were also described within the content of both chronicles [37, 86].

When describing the flora found in Maranhão, d’Abbeville and d’Évreux cited elements typical of their European cultural backgrounds as references for describing botanical species. Although with some inconsistencies, the medicinal plant descriptions included morphological aspects such as leaf shape, fruit and flower coloration, as well as references to odors, tastes, and exudates. In describing the potential of the cajú-été (*Anacardium occidentale* L.) which in the Tupi language means fruit of great value and preciousness [44, 87], Claude d’Abbeville drew morphological analogies between the pseudofruit and the pear (*Pyrus communis* L.), a species native to the European continent [53], and compared the fruit to a sheep’s kidney [24, 25]. Similarly, he related the morphological characteristics of jacarandá (*Dalbergia nigra* (Vell.) Allemão ex Benth) to those of the plum (*Prunus* sp.), and the leaves of cumaru-uaçu (*Dipteryx odorata* (Aubl.) Forsyth f.) to those of the mulberry (*Morus* sp.), describing its fruits as being approximately the size of a closed fist.

When referring to almecega (*Protium heptaphyllum* (Aubl.) Marchand), Yves d’Évreux noted similarities with typical European trees, and added to his account his own testimony regarding the uses and properties of those species [26]. This descriptive characteristic places the missionaries’ reports as foundational documents for scientific accounts, travel literature, and writings of naturalists and explorers of the 18th and 19th centuries interested in Brazilian flora [88–91].

These accounts also reveal close observations of Tupinambá customs and habits and include descriptions of indigenous cosmologies and their practices regarding the uses of petun (*Nicotiana tabacum* L.). Tupí phrases and words related to plants highlight how the missionaries perceived and portrayed cultural meanings within the Tupinambá society, incorporating indigenous knowledge into their narratives and documenting a body of traditional knowledge [2, 92, 93]. Based on their own observations, and on information shared by indigenous peoples, the missionaries conducted a type of “autopsy” a detailed examination of nature, specifically of the Brazilian flora. Although marked by exoticisms and simplistic comparisons lacking rigorous scientific descriptions, they named, documented, and attributed meanings to the “unknown” plants, providing their readers with descriptions and “demarcation(s) of similarities” to things familiar to Europeans, using a common vernacular rather than formal botanical nomenclature [34, 88, 94, 95].

**Table 1:** Medicinal plants described by Claude d’Abbeville in the *History of the Mission of the Capuchin Fathers on the Island of Maranhão and Surrounding Lands* [24] and by Yves d’Évreux in the *Journey to Northern Brazil made in the years 1613 to 1614 by Father Ivo d’Évreux* [26], organized according to the order in which they appear in those texts

Missionary	Medicinal plants	Translated Citation	Family/Species
Claude d’Abbeville	Cajú-été (Caju grande)	Caju-été, quite similar to the pear in shape; yellow on the outside when ripe. The inner part is white, full of a very sweet and pleasant juice; it is a fruit of excellent flavor. It has an outer nut shaped like a sheep’s kidney, contained in a kind of shell similar to our large chestnuts, but much harder and more porous inside, and very oily; therefore, when brought to the fire, it burns as if filled with gunpowder. The oil from this nut is good for treating dartros*; inside there is a seed that is very stomachic and as tasty as almonds.	Anacardiaceae *Anacardium occidentale* L.
	Yacarandá (Jacarandá)	The jacaranda resembles the plum tree, except that its leaves are a bit broader. The flowers are white, and the fruit, about the size of two fists, is edible, especially when cooked. The indigenous people use this fruit to make manipoí, a kind of excellent soup that is very stomachic and nutritious; the seed is about the size of a peach.	Fabaceae *Dalbergia nigra* (Vell.) Allemão ex Benth.
	Cumaru-uaçu	A large and thick tree with leaves resembling those of the mulberry, and has yellowish flowers; the fruit is a nut the size of a fist, inside of which are two or three seeds similar to white almonds; they are very fragrant and medicinal. The indigenous people use them against fever, dissolving them in water.	Fabaceae *Dipteryx odorata* (Aubl.) Forsyth f.
	Comaru-miry	It closely resembles the cherry tree, with flowers similar to those of the peach tree; the fruit is a nut the size of a white peach, containing five to six very good and medicinal kernels inside.	Fabaceae *Dipteryx alata* Vogel
	Usenpopuytan	There is another root called Usenpopuytan, which is red and, like the others, is used to make the indigenous people’s bread flour. It is very light, stomachic, and easy to digest.	Not identified
Yves d’Évreux	Petun	They always carry the herb petun (tobacco or smoke) in their mouths, the smoke of which they expel through the mouth and nostrils with the intention of drying the moisture in the brain, and at times they swallow it to cleanse the stomach of impurities, which are then expelled through belching. As soon as they finish eating, they smoke petun, and they do the same in the morning and at night, when they wake up and before going to bed. [...] They believe this herb makes them wise, judicious, and eloquent, so before beginning any speech, they make use of it. It does not seem to me to be particularly superstitious, for there is a natural explanation: I tried it myself and found that its smoke clarifies the mind by dispersing the vapors from the brain’s organs, strengthens the voice by drying the mouth’s moisture and phlegm, and thus facilitates the proper functioning of the tongue. [...] When infused for 24 h, this herb is quite effective in purifying the body of infections. Only the liquid is used. They also believe that by swallowing the smoke, they become cheerful, lively, and protected against sadness and melancholy. [...] The natives condemned to death never face execution without first using petun, in accordance with the customs of the land, and not even the sick abandon this habit. The sorcerers of the region also make profitable use of this plant, although I will not describe that now, but rather reserve it for later, if I do not forget.	Solanaceae *Nicotiana tabacum* L.
	Almecega	I saw a type of resin, similar to almecega, being extracted from the bark of a certain tree resembling the kind that grows in European gardens and that the natives say serves to treat all ailments, and thus they use it. They also say that all wild animals, when wounded or ill, turn to this tree to heal themselves, and for that reason, it is rare to find a single tree with its bark intact, as it is constantly gnawed by all sorts of creatures.	Burseraceae *Protium heptaphyllum* (Aubl.) Marchand
	Mandioca	When these natives find themselves gravely ill and are considered by their relatives to be in danger of death, they are asked what they would like to eat before dying, and their wishes are fulfilled. While sick, they feed on manioc flour and ionker (“Indian pepper”), mixed with salt, believing that with such a diet, an unheard of indulgence among them, they will recover their former health.	Euphorbiaceae *Manihot esculenta* Crantz
	Ionker (pimenta da índia)		Piperaceae *Piper nigrum* L.

*Dartros: Skin disease [55, 56]

Plants from a total of six botanical families were recorded in the chronicles, with Fabaceae being the most representative (*n* = 3). Fabaceae comprises 800 genera and more than 20,000 species, and is considered one of the three largest families in the plant kingdom in the world. The taxon is notable for its wide morphological diversity, and its native and endemic species are widely distributed across all Brazilian biomes, especially in the Amazon. The family is important for its economic, medicinal, and food uses [96, 97]. Recent ethnobotanical studies of medicinal plants conducted in the northeastern region of Brazil, particularly in Maranhão, identified Fabaceae species as the most predominant [73, 75, 98].

The majority of the species mentioned in the chronicles are native to Brazil (*n* = 6) [52], highlighting the biodiversity of that country as a key element in the process of domesticating native plant resources by indigenous populations. These species were cultivated, gathered, and selected for use in health–illness contexts and for nutrition purposes [99–101].

Non-native plants make up part of the repertoire of species used in the New World, such as black pepper (*P. nigrum*), as well as plants having their centers of distribution in the Americas, such as tobacco (*N. tabacum*). These species were incorporated into indigenous cultures through exchanges and interethnic interactions among different native populations on the American continent, as well as through the intense movement of useful plants through European colonization and circumnavigations [102–105]. As in other regions of Brazil, contact and the exchange of both information and goods, including plants, between Europeans and the Tupinambá of Maranhão had initiated in the late 16th and early 17th centuries through contacts with navigators, explorers, pirates, and merchants [106–108].

Two species reported in the chronicles, *D. nigra* (Yacarandá/Jacarandá) and *D. alata* (Comaru-miry) are currently listed as vulnerable [109]. In the case of *D. nigra*, the status is justified by excessive economic exploitation since the beginning of colonization and destruction of its natural habitat and the absence of replanting and restoration strategies in primary forests [45, 110–114]. The vulnerability of *D. alata* is due to a population decline of between 30% and 50%, as a result of the destruction of the Cerrado biome, deforestation, agricultural expansion and fires, where the species is widely found [113, 115–117].

The ongoing processes of environmental degradation in Brazil have resulted in considerable biodiversity losses [118–121]. The state of Maranhão, which encompasses phytogeographic domains characteristic of the Amazon and Cerrado, is affected by problems such as wildfires and deforestation, compromising its vegetation cover and reducing the abundance of plant species [122, 123].

The potential extinction of these species threatens the dynamics of socio-environmental systems can disrupt local communities and their biocultural heritage. Socio-environmental systems are characterized by a wealth of knowledge of plant uses as well as their management and domestication [124]. It is becoming essential to develop conservation strategies that consider diverse worldviews and traditional knowledge systems, that can generate more inclusive and effective environmental planning [125, 126].

Among the medicinal plants identified in the chronicles, medicinal indications were mentioned for the treatment of digestive problems, dermatological conditions, infections, mood disorders, wounds, and various diseases. Regarding the plant organs used, these include aerial, underground, reproductive, and vegetative structures, which are prepared by various methods, including combustion and the extraction of active principles in soluble media (such as water and vegetable oils) (Table 2).

**Table 2:** Species, medicinal indication, plant part used, traditional use, and other uses of the species as described by Claude d’Abbeville [24, 25] and Yves d’Évreux [26]

Medicinal plant/ Species	Medicinal indication	Plant part	Tradicional use
Almecega/ *Protium heptaphyllum* (Aubl.) Marchand	All ailments (Humans and other animals); Wounds	Resin	Not mentioned
Cajú-été (caju grande)/ *Anacardium occidentale* L.	Dartros; Stomachic	Fruit	Oil
Comaru-miry/ *Dipteryx alata* Vogel	Medicinal	Seed	Not mentioned
Cumaru-uaçu/ *Dipteryx odorata* (Aubl.) Forsyth f.	Antipyretic	Seed	Ingestion of powder dissolved in water
Ionker (pimenta da Índia)/ *Piper nigrum* L.	Restore health	Fruit	Condiment
Mandioca/ *Manihot esculenta* Crantz	Restore health	Root	Flour preparation
Petun/ *Nicotiana tabacum* L.	Purify the body against infection; Against sadness and melancholy; Stomach cleansing	Leaf	Smoke; Wine; Infusion
Yacarandá, Jacarandá/ *Dalbergia nigra* (Vell.) Allemão ex Benth.	Stomachic; Nutritional	Fruit	Soup
Usenpopuytan / Not identified	Stomachic; Nutritional	Root	Flour preparation

The Tupinambá, along with other indigenous ethnic groups, have developed an extensive knowledge of the tropical flora through daily interactions with their environment, applying those experiences to both to the treatment of health-related conditions and to magical-religious practices [127, 128].

The uses of cajú-été (*A. occidentale*) are diverse and consistent with the medicinal applications recorded by d’Abbeville [24, 25]. In Maranhão, indigenous groups such as the Ka’apor of Alto Turiaçu use this species to prepare ritual beverages [70], while the species is employed in the Araribóia indigenous territory to treat diabetes [61]. Experts from quilombola (Afro-descendent) communities in various regions of Maranhão have highlighted the multiple uses and therapeutic applications of the cashew tree in traditional health systems. Cashew bark and leaves are used in the preparation of “garrafadas” (bottled remedies) and decoctions, to treat inflammation and bone and muscle pain [62, 66, 74]. Other medicinal indications, such as for treating pneumonia, anemia, and wound healing, have been reported in ethnobotanical studies carried out in other quilombola territories [73, 75, 77] .

Other social groups in Maranhão also use various parts of the cashew tree to treat illnesses. The bark, leaves, and flowers are often employed to prepare teas that assist in managing diabetes, heart complications, cancer, wound healing, digestive disorders, thrombosis, respiratory diseases, and general pain [63–65, 67, 68, 71, 72, 76, 78, 79].

According to d’Abbeville [24, 25], the seeds of cumaru-uaçu (*D. odorata*) were used by the Tupinambá indigenous people to treat fevers. Among the Guajajara people of the Olho d’Água village in southern Maranhão, they are used to relieve congestion [60], while farmers, pais-de-santo (Afro-Brazilian religious leaders), benzedores (spiritual healers), and curandeiros (traditional practical healers) in the cities of São Luís, São José de Ribamar, Paço do Lumiar, and Caxias, use cumaru-uaçu seeds to treat digestive and respiratory ailments [59].

Yves d’Évreux provides an extensive description of the medicinal uses of petun (*N. tabacum*), emphasizing experimentation as a strategy to validate indigenous knowledge and the plant’s properties, describing what could be considered the stimulant effects of nicotine [26, 129]. In the early centuries of colonization, missionaries and travelers described natural resources based on observation and on personal experimentation as a way to organize and legitimize their writings [94]. The use of this species for respiratory problems has also been recently reported by residents of São Luís, the capital of Maranhão [80].

The account of d’Évreux [26] describes the resin of almecega (*P. heptaphyllum*) as being used as a treatment for wounds and for many ailments affecting both humans and animals. The resin exuded by this species has a long history of medicinal use in Brazil [130, 131].

Friar Francisco de Nossa Senhora dos Prazeres (1790–1852), in his work *Poranduba Maranhense* [58] describes almecega as a *“[…] small tree that produces the drug bearing its name. It is rarely seen”.* Its resin, when ground and mixed with tobacco leaves for use as snuff, has been used to treat respiratory problems in northeastern Brazil, from Maranhão to Bahia [132]. Berg and Silva [59], in their study of the medicinal flora of Maranhão, highlighted the use of the leaves of this species to prepare teas for treating respiratory complications. Its roots, barks, and resin are also used by traditional communities in Maranhão to address health issues such as inflammation and digestive problems [62, 69, 75].

Yves d’Évreux [26] reported the consumption of flour made from mandioca (*M. esculenta*) by the Tupinambá as a food supply. According to the missionary, the flour was prepared with ionker or pimenta da índia (*P. nigrum*) and administered to aid in the recovery of ill individuals. This practice highlights manioc as a primary source of subsistence, with symbolic and functional value within the context of indigenous food security [133, 134]. The uses and domestication of this plant continue to be the food basis of several Amazonian peoples and communities to this day [135]. In the 20th century, Rêgo [49] noted its use in Maranhão to treat intestinal problems.

The descriptions provided by the Capuchin missionaries reveal the medicinal potential of species and reinforce the observation that indigenous peoples had therapeutic knowledge [136]. Food has an intrinsic relationship with the Tupinambá indigenous medical system. In this sociocultural context, food and nutrition represent a medicinal interface of plants [137–139].

Five of the eight medicinal plants cited in the chronicles of Claude d’Abbeville [24, 25] and Yves d’Évreux [26] show continuity, similarity, or expansion of their medicinal uses as cited in other accounts and publications from the 19th [58], 20th [49, 59, 60] and 21st centuries from Maranhão [61–80] (Table 3). Botanical information has been shared and hybridized among missionaries and indigenous peoples through transcultural contacts dating back to the colonial period, with knowledge being gradually incorporated and produced by new cultures, over the centuries, to the present day [31, 140]. A socially constructed and living memory of a knowledge system in constant transformation is created [33, 40, 141–143].

**Table 3:** Timelessness of the medicinal uses of species cited in the chronicles of the Capuchin missionaries Claude d’Abbeville [24, 25] and Yves d’Évreux [26] through a diachronic analysis spanning the 19th, 20th, and 21st centuries in the state of Maranhão (Brazil)

Species	19th century	20th century	21st century
*Anacardium occidentale* L.	-	Antifungal, antisyphilitic, healing (wound healing), diarrhea, athlete’s foot [49, 59]	Cancer, wound healing, heart complications, diabetes, musculoskeletal diseases, respiratory diseases, general pain, digestive system disorders, pneumonia, thrombosis, and inflammation treatment [62, 64, 65, 67, 68, 70, 72–79]
*Dalbergia nigra* (Vell.) Allemão ex Benth.	-	-	-
*Dipteryx alata* Vogel	-	-	-
*Dipteryx odorata* (Aubl.) Forsyth f.		Congestion, digestive and respiratory problems [59, 60]	Medicinal [75]
*Manihot esculenta* Crantz	-	Intestinal ailments [49]	Eye conditions, malnutrition [77]
*Nicotiana tabacum* L.	-	-	-
*Piper nigrum* L.	-	-	Respiratory diseases [80]
*Protium heptaphyllum* (Aubl.) Marchand	Medicinal [58]	Asthma [59]	Respiratory diseases, digestive diseases, inflammations, medicinal uses [62, 69, 75]

**Fig. 4:**
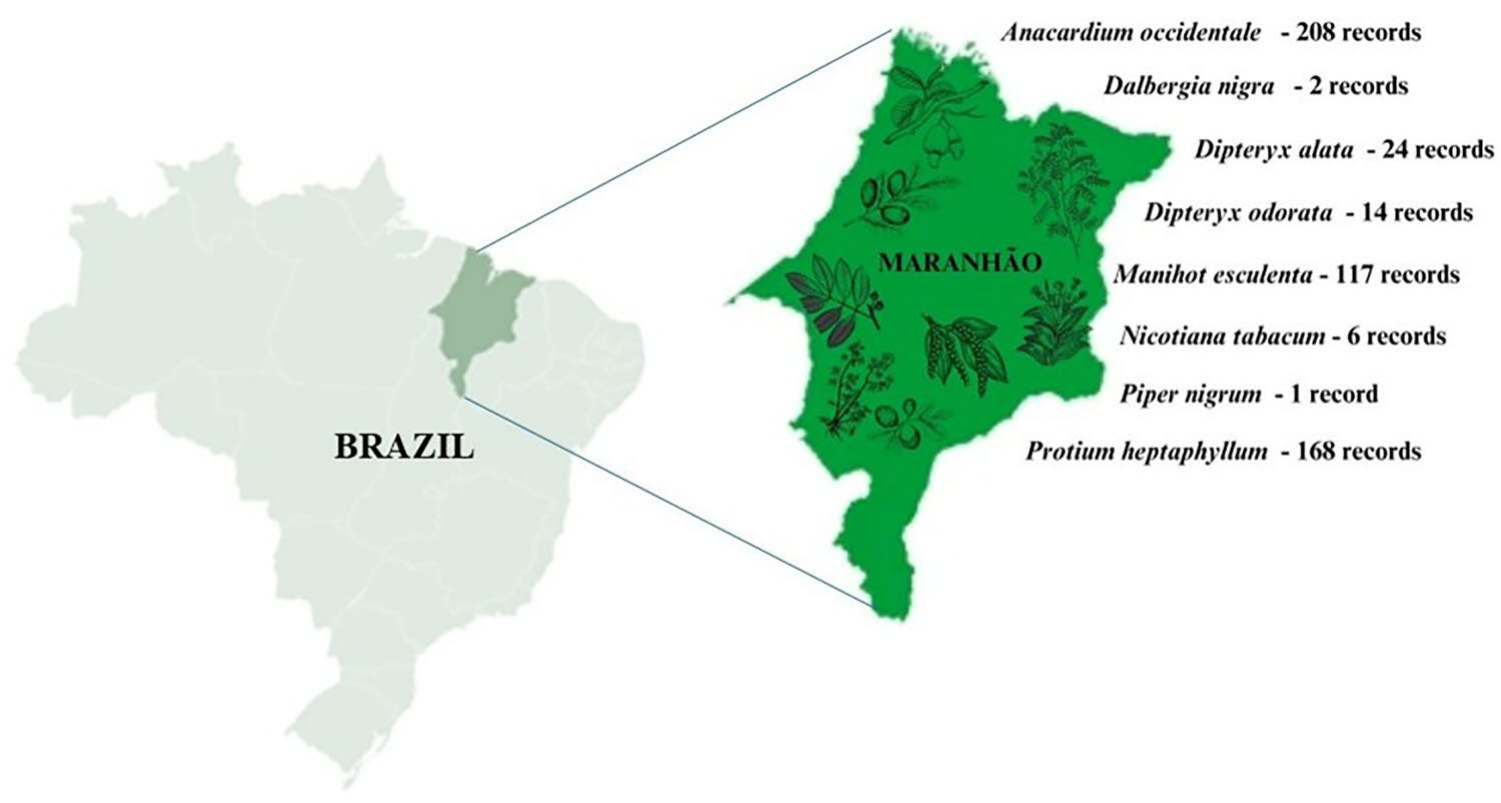
Records of medicinal specimens used by the Tupinambá according to the Capuchin missionaries d’Abbeville [24, 25] and d’Évreux [26] collected in the state of Maranhão (in northeastern Brazil) consulted through SpeciesLink [81] and GBIF [82]

The entire set of medicinal plants cited by d’Abbeville and d’Évreux [24–26] have since been collected in the state of Maranhão, but among all of the specimen collections in herbaria (*n* = 540; Fig. 4), only a small subset (*n* = 16; 2.82%) have annotations of cultural/medicinal uses. The insufficiency of ethnobotanical data in those herbarium records highlights how such information is often overlooked, despite being fundamental for the documentation of biocultural knowledge [144, 145]. Consultations of these collections demonstrate the richness and biological diversity of the local flora, helping to safeguard knowledge that has been both preserved and transformed over time, and reveal links between humans and plants, and the cultural and historical relationships surrounding the use and domestication of species [146, 147].

In the search for records of medicinal uses, two species cited by d’Abbeville and d’Évreux [24–26] were found with medicinal uses noted on the labels of exsiccate of specimens deposited in the herbaria New York Botanical Garden (NY), and the Field Museum of Natural History (F) (Table 4).

**Table 4:** Records of specimens and information cited on plant exsiccatae collected in the state of Maranhão (Brazil) and available from the SpeciesLink [81] virtual herbaria referring to medicinal plants mentioned in the chronicles of the Capuchin missionaries d’Abbeville [24, 25] and d’Évreux [26]

Species	Popular name	Traditional uses	Collection site	Collector Herbarium Collection date
*Dipteryx odorata* (Aubl.) Forsyth f.	Cumarú	Kernels used for oil and for heart diseases	Campo de Boa Esperanca, Maracassumé River region, Cururupu Maranhão, Brazil	R. de Lemos Fróes NY 05/09/1932
	Kumaru’y	Oil from seeds put into ears to cure earaches	Basin of the Rio Turiaçu; Ka’apor Indian Reserve; within 7 km of the settlement of Urutawy, Monção Mun., Maranhão, Brazil	W. L. Balée NY 05/06/1985
*Protium heptaphyllum* (Aubl.) Marchand	Almesca	Resin used as a cure for headaches	Maracaçumé Maranhão Brazil	R. Fróes F 14/9/1932

**Fig. 5:**
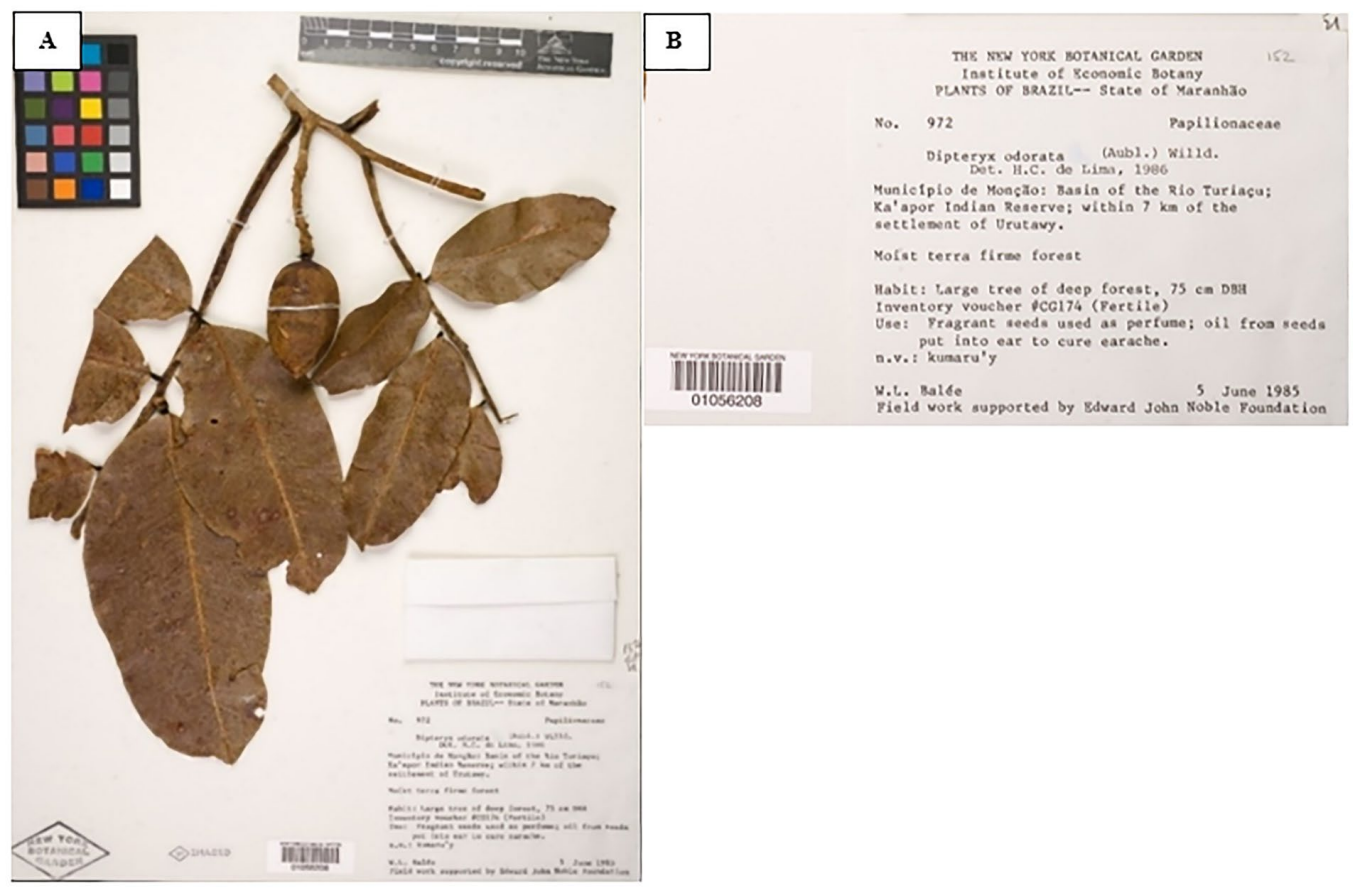
(**A**) Specimen of *Dipteryx odorata* (Aubl.) Forsyth f. collected in Maranhão by W. L. Balée on June 5, 1985, identified by H. C. de Lima in 1986. Deposited at The New York Botanical Garden. (**B**) Details from the label describing its uses. SpeciesLink [81]

These records highlight the therapeutic knowledge of the use of *D. odorata* resin for the treatment of heart problems and earaches (Fig. 5A, B) and the use of *P. heptaphyllum* resin for the relief of earaches. These indications, grounded in traditional knowledge of plant biodiversity, are fundamental to the advancement of ethnobotanical research [148]. The collections and information from the 20th and 21st centuries, as documented on specimen labels, strengthen records of the occurrence of these medicinal plants in the territory of Maranhão, as originally described by d’Abbeville and d’Évreux in relation to the Tupinambá people in the 17th century.

## Conclusions

Based on a “hybrid” intellectual exchange between the missionaries Claude d’Abbeville and Yves d’Évreux and the Tupinambá indigenous people, historical ethnobotany was employed to analyze the historical memories transcribed in the chronicles that emerged from this Franco-Tupi relationship. By correlating vernacular names described by the Capuchins referring to plants used by the Tupinambá during the 17th century in the territory of Maranhão, Brazil, it was possible to infer an indigenous contribution to the knowledge of the use of medicinal plants.

Virtual herbarium collections revealed that the entire set of medicinal species described hundreds of years earlier (17th century) still has registers of collect in the 20th and 21st centuries in that state, although some are vulnerable in the wild, and ethnobotanical information is often neglected on specimen labels.

It is hoped that future studies of these plants will deepen discussions concerning their conservation both in terms of their biocultural heritage and in relation to health care. The historical investigation of human interactions with plant resources through documentary evidence can rekindle, contribute to, and help preserve traditional knowledge.

## Supplementary Information

Below is the link to the electronic supplementary material.

Supplementary Material 1

## Data Availability

No datasets were generated or analysed during the current study.
