# Supplementary material for: The Tupinambá of Maranhão State, Brazil, and medicinal plants described in the 17th century chronicles of the Capuchins Claude d’Abbeville and Yves d’Évreux
Source: J Ethnobiol Ethnomed. 2026 May 3;22:69. doi: 10.1186/s13002-026-00872-x (PMC13285183; doi:10.1186/s13002-026-00872-x)
Supplement: Supplementary file 1 — Supplementary Material 1 [file 13002_2026_872_MOESM1_ESM.docx]

**SUPPLEMENTARY FILE**

Descriptions of medicinal plants recorded in the works of Claude d’Abbeville (Histoire de la mission des Pères Capucins en l'Isle de Maragnan et terres circonvoisines/History of the mission of the Capuchin Fathers on the Island of Maranhão and its surroundings) and Yves d’Évreux (Voyage dans le nord du Brésil fait durant les années et 1613 et 1614 par le Père Yves d’Évreux/Journey to Northern Brazil undertaken in the years 1613 to 1614 by Father Ivo d’Evreux) in the original French version and in the Portuguese version.

| Missionary | Medicinal Plants/ Original French version | Medicinal Plants/ Portuguese version |
| --- | --- | --- |
| Claude d’Abbeville | Acaiou été / qui reſſem- ble aſſez à la poire quant à ſa forme & eſt tout iaulne au dehors lors qu'il eſt en ſa maturité. Le dedans eſt tout blanc remply de ſuc fort doux & agreable, eſtant vn fruit tres excellent à manger. Il porte vne noix à ſon œuiller en dehors, laquelle eſt de meſme façon qu’vn roignon de Mouton, ayāt vne coquille vnie par le deſſus comme celle d’vn Marron, mais beaucoup plus dure & porreuse par le dedās & aucunement huileuſe; qui fait qu’eſtant alumée au feu elle brule comme ſi elle eſtoit pleine de feu artificiel. L’huile prounant de cette coquille eſt fort ſinguliere pour les dartres: au dedās d’icelleil ſe trouue vn noyau fort stomacal, non moins excellent que les Amandes (p. 217-218, 1864). | Cajú-été (Caju grande) / *Muito parecido com a pera, e quando maduro é todo amarello por fóra, e branco por dentro, muito doce e agradavel, e optimo para se comer. Tem uma castanha muito parecida com o rim do carneiro, a qual está contida n'uma concha, muito similhante a uma das nossas castanhas grandes, porem muito mais dura por dentro, e oleosa, e por isso chegando-a ao lume arde como se estivesse cheia de fogo artificial. Este oleo é muito bom para dartros. Contem uma noz muito estomacal, e tão boa como as amendoas.* (p. 252, 1874). |
|  | Yacaranda / eſt ſemblable au prunier,excepté que ſes fueilles ſont vn peuplus larges les fleurs en ſont blanches & le fruit auſſi gros que les deux  poings, il eſt bon à manger principalement quand il eſt cuit. Les Indiens ſe ſeruent de ce fruit pour faire du Manipoy qui eſt vne eſpece de potage fort excellent à manger, bien ſtomachal & nutritif : ce fruit a vn noyau gros comme la peſche (p.223, 1864). | Yacarandá (Jacarandá) / *Similhante a ameixieira, menos nas folhas, que são um pouco mais largas: suas flores são brancas, e seu fructo da grossura de dous punhos cerrados, é muito bom para comer mormente cozidos. D'estes fructos se servem os Indios para o fabrico do Manipoy, sopa muito boa, muito estomacal e nutrictiva: tem dentro uma amendoa do tamanho de um pecego.* (p. 258, 1874).  **Tabela 1** (continuação)  **Tabela 1** (continuação)  **Tabela 1** (continuação) |
|  | Comaron Ouäſſou / eſt grand & gros, ayāt la fueille aſſez ſemblable au Meurier & la fleur iaunaſtre: ſon fruit eſt comme vne noix de la groſſeur du poing le quel eſtant rompu, l’on y trouue deux,trois ou quatre noyaux, comme les  groſſes Amandes ils ſont fort odoríferas & medicinaux: les Indiens s’en ſeruent pour ſe guarentir de la fieure le beu-uant en poudre avec de l’eau (p.226, 1864). | Cumaru-uaçu */ Grande e grossa, com folhas parecidas com as da amoreira, e flores amarellas. Seu fruto tambem é uma noz do tamanho de um punho, contem duas, tres e quatro amendoas grades ordoriferas e medicinaes, e os indios redusem-nas a pó, dissolvem este n'agua, e bebem como remedio anti-febril.* (p.261, 1874). |
|  | Comarou miry eſt aſſez ſemblable au Ceriſier; ayāt ſa fleur comme celle du Peſcher: ſon fruit eſt vne noix comme vne groſſe Peſche, il le faut rompre pour auoir cinq ou ſix grains qui ſont  dedans fort bons & medicinaux.(p.226, 1864). | Comaru-miry / *Parece-se muito com a cereja, e tem flores iguaes ao pecegueiro. Seu fructo é do tamanho de um pecego, porem elos maiores, como uma noz, e sendo quebrada encontra-se cinco ou seis grãos muito bons medicinaés.* (p. 261, 1874) |
|  | Vſenpopoytan/ laquelle eſt rouge, & eſt fort propre comme les autres pour faire de la farine, dont les Indiens vſent ordinairement au lieu de pain, eſtant vne nourriture fort ſtomachale, legere  & de facile digestion (p.230, 1864). | Usenpopuytan */ Outra raiz muito vermelha, e propria como as outras para o fabrico de farinha, de que usão os indios ordinariamente em lugar de pão, sendo uma nutrição mui estomacal, ligeira e de facil digestão.* (p.265, 1874). |
| Yves d’Évreux | Petun / Ils ont tousjours l’herbe de Petun en la bouche, la fumee de laquelle ils attirent par la bouche, & le rendent par les narines, afin de vuider les humiditez du Cerneau, & en aualent, pour nettoyer l’estomach de cruditez, lesquelles ils font sortir par eructations. Ils n’ont pas si tost acheué de manger qu’ils prennent leur Petun, comme ils font aussi du grand matin, àla sortie du lit, & auant de se coucher. Mais à propos du Petun, il est bon que ie rapporte icy l’opinion superstitieuse qu’ils ont de cette herbe, & desa fumee. Ils croyent que cette fumee les rend diserts, de bon iugement & eloquens en parole, tellement que iamais ils ne commencent vne harangue qu’ils n’en ayent pris. Et me semble que leur opi nion n’est point tant superstitieuse, qu’elle n’aye quelque raison naturelle; car ie l’ay experimenté moy mesme, que cette fumee esclairecit l’entendement, dissipant les vapeurs, qui possedent l’organe du Cerneau, & affermit la voix, en ce qu’elle desseiche les humiditez & crachats de la bouche, qui se rencontrent à la sortie de la veine vocale tellement que la langue en est bien plus libre à faire sa fonction: La verité de cecy est bien aisée à experimenter,pourveu qu’on en prenne avec modestie, & au temps conuenable: Carl’abondance & continuation n’en est pas, à mon aduis, trop bonne & salubre à ceux qui viuent de boissons & viandes chaudes ; mais à ceux qui sont humides & froids de cerueau & d’estomach, la prise de ceste fumee ne leur peut estre que saine ; Et c’est vne autre raison, pourquoy les Sauuages qui habitent sous cette zone tres-humide, & qui pour l’ordinaire ne boiuent que de l’eau, prennent continuellement de ceste fumee, à sçauoir pour deschar-er leur Cerueau des humiditez & froidures, & l’estomach de cruditez: ce que font semblablement lesMatelots & les gens habitans sur le riuage de la mer. Ce Petun aussi ayans trempé 24. heures dans du vin blanc, opere de grands effects pour nettoyer le corps de ses infections. On ne prend seulement que levin. Ils ont aussi vne autre opinion que la fumee qu’ils aualent du Petun, les tient gaillards & ioyeux contre la tristesse & melancholie qui leur peut suruenir. Ie vous le feray voir par exemples, outre ce que j’en ay peu apprendre par leurs discours (p.110, 1864). | Petun */ Trazem sempre na bocca a herva do Petun, (tabaco ou fumo) cujo fumo expellem pela bocca e narinas com intenção de seccar as humidades do cerebro e as vezes o engolem para limpar o estomago de cruezas que saem por meio do arrôto. Apenas acabam de comer fumam o Petun, e o mesmo praticam pela manhan e a noite, quando se levantam e deitam- se. [...] Creem, que esta herva os torna discretos, judiciosos, e eloquentes, de forma que antes de começarem algum discurso usam d’ella: não me parece, que seja comtudo muito supersticiosa, porque ha nisto uma razão natural: eu mesmo a experimentei, e reconheci, que a sua fumaça exclarece o entendimento dissipando os vapores dos orgãos do cerebro, fortalece a voz seccando a humidade e escarros da bocca, permitindo assim facilidade á lingua para bem exercer suas funcções. [...] Pondo-se de infusão por espaço de 24 horas esta herva, presta-se muito para purificar o corpo de infecções. Usa-se somente do vinho. Creem também que, engolindo o fumo, ficam alegres, joviaes e previnidos contra a tristesa e melancolia. [...] Os selvagens sentenciados á morte não soffrem a pena sem usarem antes do Petun, conforme o costume da Terra, e não deixavam este habito nem mesmo os doentes. Os feiticeiros do paiz servem-se d’esta planta com proveito, o que agora não refiro, e sim guardo para o fazer mais adiante, si não me esquecer.* (p. 99 – 100, 1874) |
|  | I’AY veu de l’escorce d’vn certain arbre, la quelle sentoit tout ainsi que le Mastic, qui croist aux Iardins de l’Europe, & les Sauuages disent que ceste escorce sert à toute maladie, & en vſent : Dauantage ils tiennent que toutes les bestes des forests, se sentans ou frappees ou malades, courent à cet arbre pour auoir guerison: & pour cette cause rarement peut on trouuer vn de ces Arbres qui aye l’escorce entiere, parce que les bestes & animaux du pays la viennent ronger (p.118, 1864). | Almecega */ Vi tirar-se da casca de certa arvore uma especie de almecega, similhante á que cresce nos jardins da Europa, e dizem os selvagens que serve para toda a molestia, e assim a empregam. Contam mais, que todos os animaes ferozes quando se sentem feridos ou doentes, recorrem a esta arvore para curarem-se, e por isso raras vezes se encontra uma só com toda a sua casca, por ser roida constantemente por todos os bixos.* (p. 106, 1874) |
|  | Manioch, Ionker, du pouire d’Inde / CES Sauuages atenuez de maladie, depuis qu’ils ſont iugez à mort par leurs parens, on leur demande ce qu’ils deſirent de manger auant que de mourir, & auſſi toſt il leur eſt trouué: combien que leurs repas ordinaires, tandis que la maladie dure, ne ſoient autres, que de la farine de Manioch, & du Ionker, c’eſt-à-dire du poivre d’Inde, meſlé avec le ſel: croyans que par ceſte diſette, ils recouureront leur priſtine ſanté, qui eſt vn grand abus entr’eux: car i’ay veu moy-meſme vn homme & vne femme de la nation des Tabaiares, qui n’auoient que les os & la peau, & à noſtre iugement ils ne pouuoient viure encore deux iours, (& toutesfois pour cet effet, les baptiſans apres l’auoir requis) que leur ayant faict prendre de bons boüillons, ils eſchaperent pour ceſte fois la mort (p.125, 1864). | Mandioca, Ionker (pimenta da índia)  */ Quando se acham muito doentes estes selvagens, e por seos parentes julgados em perigo de vida, perguntam-lhes o que desejam comer antes da morte, e saciam-lhes o desejo. Em quanto doentes alimentam-se com farinha de mandioca e ionker «pimenta da india,» misturada com sal, julgando com tal dieta, abuso inaudito entre elles, recobrarão a antiga saude.* (p.112, 1874) |

**References**

D’Abbeville C. Histoire de la mission des Peres Capucins en l’Isle de Maragnan et terres circonuoisines, où est traicté des singularitez admirables et des moeurs merveilleuses des Indiens habitans de ce pays, avec les missives et advis qui ont esté envoyez de nouveau pa [Internet]. França: Biblioteca Nacional da França; 1614. <https://gallica.bnf.fr/ark:/12148/btv1b86057861?rk=42918>

D’Évreux Y. Voyage dans le Nord du Brésil, fait durant les années 1613 et 1614 / par le Père Yves d’Évreux. A. Franck (Leipzig), editor. França: Librairie A. Franck; 1864. <https://gallica.bnf.fr/ark:/12148/bpt6k5732857p>

D’Abbeville C. História da missão dos padres capuchinhos na ilha do Maranhão e suas circumvisinhaças [Internet]. Typ. do Frias; 1874. <https://www2.senado.gov.br/bdsf/handle/id/221724>

D’Evreux I. Viagem ao Norte do Brasil Feita nos Anos de 1613 a 1614, Pelo Padre Ivo D’Evreux [Internet]. Marques CA, editor. Maranhão: typ. da Frias; 1874. <https://digital.bbm.usp.br/view/?45000030119&bbm/7866#page/1/mode/2up>
